# Level Set Method for Positron Emission Tomography

**DOI:** 10.1155/2007/26950

**Published:** 2007-06-25

**Authors:** Tony F. Chan, Hongwei Li, Marius Lysaker, Xue-Cheng Tai

**Affiliations:** ^1^Department of Mathematics, University of California, Los Angeles, 405 Hilgard Avenue, Los Angeles, CA 90095-1555, USA; ^2^Center for Integrated Petroleum Research, University of Bergen, CIPR room 4103, Allégaten 41, 5007 Bergen, Norway; ^3^Department of Scientific Computing, Simula Research Laboratory AS, 1325 Lysaker, Norway; ^4^Department of Mathematics and System Sciences, Henan University, Kaifeng 475001, China; ^5^Department of Mathematics, University of Bergen, Johannes Brunsgate 12, 5009 Bergen, Norway

## Abstract

In positron emission tomography (PET), a radioactive compound is injected into the body to promote a tissue-dependent emission rate. Expectation maximization (EM) reconstruction algorithms are iterative techniques which estimate the concentration coefficients that provide the best fitted solution, for example, a maximum likelihood estimate. In this paper, we combine the EM algorithm with a level set approach. The level set method is used to capture the coarse scale information and the discontinuities of the concentration coefficients. An intrinsic advantage of the level set formulation is that anatomical information can be efficiently incorporated and used in an easy and natural way. We utilize a multiple level set formulation to represent the geometry of the objects in the scene. The proposed algorithm can be applied to any PET configuration, without major modifications.

## 1. INTRODUCTION

**Figure 1:**
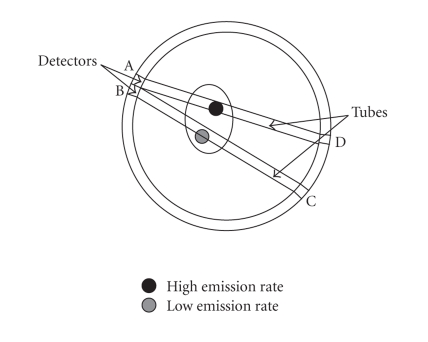
Gamma rays escape the body and are observed by the detectors.

One of the most important quality of PET is its abilities to model biological and physiological functions in vivo to enhance our understanding of the biochemical basis of normal and abnormal functions within the body. PET is also useful for the detection of cancer, coronary artery disease, and brain disease. During a PET acquisition, a compound containing a radiative isotope is injected into the body to form an (unknown) emission density *λ*(*x, y*) ≥ 0. The positron emitted finds a nearby electron and they annihilate into two photons of 511 keV according to the equation *E* = *mc* ^2^. This energy is strong enough to escape the body. Since the two photons travel at almost opposite directions, a detector ring surrounds the patient and tries to collect the emissions. For an emission event to be counted, both photons must be registered nearly simultaneously at two opposite detectors. In Figure 1, emission paths from two different regions are shown, that is, along the tube covered by detector pair AD, and along the tube covered by detector pair BC. Regions with higher concentration of radioactivity cause a higher emission rate. Given the total number of measured counts for each detector pair, the challenge is to locate all the emission sources inside the detector ring. Emissions measured between two detectors could have taken place anywhere along the tube between these two detectors, but with a systematic inspection of all detector pairs, it is possible to reveal variance in the emission rate along the same tube.

Detection of the radioactive concentration in different tissues gives useful information both for research and clinical purposes. This information is often analyzed and visualized as an image. Unfortunately, the measured events also include noise such as accidental coincidences that complicate the image reconstruction. The Fourier-based filtered back-projection [1] algorithm is a well-established construction technique. This algorithm is computationally efficient, but the drawbacks are constructions with low signal-to-noise ratio and low resolution. Iterative methods to construct PET images have been an attractive approach during the last two decades. Most of these methods are based on maximum likelihood estimates. Due to the inherent ill-posedness of this inverse problem, the reconstruction process suffers from noise and edge artifacts, see [2, 3] for related problems. It is well known that the standard EM algorithm [4–6] converges toward a noisy image and it is necessary to terminate the iteration before the noise becomes too dominant [7]. If the iteration stops too early, important information could be lost. A general approach to address these problems is to utilize a regularization term according to certain a priori assumptions of the desired image [8–10]. Results with deviation from these assumptions will be penalized. For example, information from surrounding pixels can reveal irregularities and remove outsiders. The total variation (TV) minimization has been successfully used in many image processing applications [11–17]. In [18], the standard EM algorithm for PET was modified to incorporate the TV regularization. The blurring effect was subdued by using this approach, but improvements are still needed.

Common for the iterative methods mentioned above is that they estimate the concentration coefficients that provide the best fitted solution based on a maximum likelihood estimate. In PET, different tissues should have different active levels, while the active values should change smoothly and slowly in the same tissue [19]. So, the PET image is actually a piecewise smooth function [20]. Recently, PET has been combined with CT and MRI devices [21–24]. CT and MRI can provide high-resolution structural information, which can be incorporated into the reconstruction process to improve the properties of the constructed PET image. Usually the anatomical information obtained from CT or MRI image is used as a Bayesian prior. A penalty technique is utilized to build in the Bayesian prior, while a parameter *β* to control the strength of the penalty. However, this approach is very sensitive to the penalty parameter, and finding a proper *β* can be challenging. In this paper, we use a level set method to serve this purpose. We reduce the set of possible solutions by estimating the emission rate as a piecewise constant function. This can be thought of as an approximation to the piecewise smooth image. By this way, the anatomical information based on CT or MRI can be used as the initial guess for the level set curves, without the need to estimate the penalty parameter *β*. We can see in the numerical experiment section that the quality of the reconstruction improves with the quality of the anatomical data.

Level set method is a versatile tool which has been applied to many areas of computational mathematics [25–29]. As in many other applications, the level set method is used here to capture the coarse scale information and the discontinuities of the function to be recovered. By incorporating the level set method into the image reconstruction, sharp boundaries between different tissues are directly obtained for PET images. This variant of the EM algorithm (called LSEM hereafter) can be applied to any PET configuration, without major modifications. We will first show that even without anatomical information available, LSEM can produce better images than EM algorithm in some sense. Moreover, LSEM can easily and naturally incorporate anatomical information (interior boundaries for different tissues, which can be obtained from CT or MRI images), and improve the quality of the reconstructed images further. It is well known that one drawback of the EM algorithm is its lack of stopping criterion. In this paper, TV regularization will be used to deal with the ill-posedness of the reconstruction process. The parameter used to weight the influence of the TV regularization more explicitly controls the tradeoff between regularity and fitting the data. There is a number of well-known techniques for choosing this parameter more systematically [18, page 6].

Geometric curve-evolution techniques for tomographic reconstruction problems have been proposed previously, see [19, 30–37] and the references therein. Similar to [31], we assume the object intensity values to be piecewise constant, but we allow for multiple object regions as in [19]. Due to the piecewise constant intensity value restriction, our cost functional is simpler than the one proposed in [19]. In [36, 37], a strategy for joint estimation of the unknown region boundaries and the unknown activity levels was developed. However, the movement of the parameterized boundaries involved only translation, rotation, and scaling. We propose a more flexible framework here, and allow multiple object regions. In addition, level set method is more flexible and efficient in dealing with complicated geometries, thanks to its great ability to handle topological changes during the curve evolution. In [38], the authors proposed a piecewise smooth model for emission tomography reconstruction, which also utilized the level set framework. Compared to that model, our piecewise constant model is simpler, and can simplify the computations. We would like to emphasize that in most cases, our method could still work well even if the intensity function is not piecewise constant. In fact, our method just needs that the object in the scene can be well segmented into several phases. We will show some results in the numerical experiment section to verify it.

The remainder of this paper is organized in the following way. In Section 2, we summarize the theory behind the EM approach and introduce some specific notations used throughout this paper. Partial differential equation techniques have been successfully used in many image processing applications, and a predecessor for our approach is given in Section 3. In Section 4, we explain the main idea behind the level set method and demonstrate that level set functions can be used to represent general piecewise constant functions [29, 39, 40]. Motivated by this, we utilize a level set formulation to represent PET images with piecewise constant emission densities in Section 5. In this section, we also give implementation details. Finally, we report some numerical results in Section 6.

## 2. MAXIMUM LIKELIHOOD EXPECTATION MAXIMIZATION

From the measured emission an image can be constructed by the EM algorithm [5, 6]. This algorithm provides an iterative formula to construct an image which makes the measured data most likely to occur. Given an image, the aim is to maximize the conditional probability of the data by using a likelihood function (and later we will also use a log-likelihood function):

(1) $$l(\lambda )=f(\text{data}\;|\;\lambda )\;\text{or}\;l_{\log}(\lambda )=\log(l(\lambda )).$$

Here, data are the measured counts in the detector ring, *λ* :Ω → ℝ is the unknown emission rate causing these counts, and *Ω* is the image domain. The region to be reconstructed is usually covered by a uniform mesh, where each square in the mesh corresponds to one pixel in the PET image. The discrete representation of *λ* and other essential notations for describing the EM image reconstruction model are listed in Table 1. To simplify the notations, we still use *λ* to denote its discretized version, for example, *λ* = (*λ*_1_, *λ*_1_…, *λ*_*B*_)^T^.

Each element *P*_*tb*_ in matrix *P* describes the probability for an annihilation event that occurred in the area of the source covered by pixel *b* to be detected by detector pair *t*. Several physical factors such as attenuation, scatter and accidental coincidence corrections, time-of-flight, positron range and angulation information, and so forth, can be incorporated in the probability matrix *P*. To compute *P*_*tb*_, the angle-of-view method was chosen in this paper, but other methods can also be used [41, 42]. By the angle-of-view method, each element *P*_*tb*_ in the probability matrix *P* is approximated by the largest angle (in fraction of *π*) completely contained within tube *t* as seen from the center of *b*. For details about the angle-of-view, see the paper of Shepp and Vardi [6]. The intensity value *λ*_b_ is the information we are seeking since it is related to the tracer concentration. During the acquisition process, a random number of emissions is generated from a Poisson distribution. A nonnegative, integer-valued, and random variable *Z* follows a Poisson distribution if

(2) $$\text{Poisson}(Z\,=k)=e^{-\sigma }\frac{\sigma^{k}}{k!},$$

where *σ* > 0 and *Z* has mean *E*(*Z*) = *σ*. The Poisson distribution is applicable to many problems involving random events, such as particles leaving a fixed point at a random angle. For the moment, we focus on one of the tubes in Figure 1 and assume that this tube corresponds to the region covered by detector pair *t* = 1. Given the mean (*P*λ**)_1_, we want to maximize the probability for (*P*λ**)_1_ to fit the measured data *n*_1_:

(3) $$\text{Poisson}(Z=n_{1})=e^{-{\left(P\lambda \right)}_{1}}\frac{{\left(P\lambda \right)}_{1}^{n_{1}}}{n_{1}!},$$

where a maximum is obtained for *n*_1_ = (*P*λ**)_1_, and similarly the maximum is achieved for *n*_2_ = (*P*λ**)_2_ if we focus on region covered by detector pair *t* = 2. The measured coincidence events also include scattered and accidental coincidences. Some events produced inside the source pass are undetected because of tissue attenuation or photon travelling path that does not intersect the detector ring. This complicates the image construction. However, each *n*_t_ is distributed according to a Poisson distribution and since all measurements are independent of each other, the likelihood over all projections reduces to the product of the separate projections likelihood [5]. Therefore we want to maximize

(4) $$l(\lambda )=\prod_{t=1}^{T}e^{-{\left(P\lambda \right)}_{t}}\frac{{\left(P\lambda \right)}_{t}^{n_{t}}}{n_{t}!}.$$

To simplify the calculation, the log-likelihood function is employed to convert (4) to the form

(5) $$\begin{array}{ll} l_{\log}(\lambda ) & =\overset{T}{\underset{t=1}{\sum }}[\log e^{-{\left(P\lambda \right)}_{t}}+\log{\left(P\lambda \right)}_{t}^{n_{t}}-\log(n_{t}!)]\\ & =-\overset{B}{\underset{b=1}{\sum }}\lambda_{b}+\overset{T}{\underset{t=1}{\sum }}n_{t}\log{\left(P\lambda \right)}_{t}+K. \end{array}$$

In (5), we assume

(6) $$\sum_{t=1}^{T}P_{tb}=1$$

for any pixel *b*, and then exploit the conversions

(7) $$\overset{T}{\underset{t=1}{\sum }}{\left(P\lambda \right)}_{t}=\overset{B}{\underset{b=1}{\sum }}\lambda_{b},\;-\overset{T}{\underset{t=1}{\sum }}\log(n_{t}!)\overset{\text{def}\text{.}}{=}K.$$

Since *K* is independent of *λ*, this constant will be ignored. Maximizing *l*_log_(*λ*) with respect to *λ* will provide us with the best estimate of *λ* in a statistical sense. The optimization problem can be rewritten by max*l*_log_(*λ*) = min(−*l*_log_(*λ*)), and thereupon a mathematical formulation of PET becomes

(8) $$\underset{\lambda }{\min}F(\lambda )=\underset{\lambda }{\min}(\sum_{b=1}^{B}\lambda_{b}-\sum_{t=1}^{T}n_{t}\log{\left(P\lambda \right)}_{t}+V(\lambda )),$$

where *V* (*λ*)is a regularization term introduced to improve image quality [7–10, 18, 43]. Several regularization methods tend to blur edges because both noise and edges contribute to inhomogeneous behavior. To subdue the blurring effect, the total variation norm of *λ* was introduced as a regularization term in [18]. In the next section, we give a short overview of the TV-based EM algorithm.

## 3. A TOTAL VARIATION-BASED EM ALGORITHM

In [18], an algorithm was designed to find the pointwise values of *λ*. The authors covered the domain Ω with a uniform mesh, where each square in the mesh corresponds to one pixel in the PET image. The emission density function *λ* is represented by a piecewise linear function or piecewise constant function where *λ* takes value *λ*_b_ at pixel *b*, *b* = 1, 2,…, *B*. In order to regularize the problem, they find a minimizer for the following functional:

(9) $$L(\lambda )=\mu \int_{\Omega }|\nabla \lambda |dx+(\sum_{b=1}^{B}\lambda_{b}-\sum_{t=1}^{T}n_{t}\log{\left(P\lambda \right)}_{t}).$$

From (9), it is easy to see that

(10) $$\frac{\partial L}{\partial \lambda }=\mu C(\lambda )\lambda +\vec{e}-P^{t}(\vec{n}./P\lambda ).$$

In the above, *C* (*λ*) is a matrix depending on *λ*, $\vec{e}$ is the vector with unit entries, *P* ^t^ is the transpose of the matrix *P*, and $\vec{n}./P\lambda$ is the elementwise division of vector $\vec{n}$ by vector *P* *λ*. In [18], the following fixed point iteration was used for finding the minimizer of (9):

(11) $$\lambda^{k+1}=[\mu C(\lambda^{k})+diag(1./\lambda^{k}{)]}^{-1}P^{t}(\vec{n}./P\lambda^{k}).$$

In (11), diag(1./*λ* ^*k*^) is the matrix with 1./*λ* ^*k*^ on its diagonal and *C*(*λ* ^*k*^) is a finite difference approximation for − ∇ · (∇ *λ* ^*k*^./∣∇ *λ* ^*k*^∣).This scheme is obtained from (10), by replacing $\vec{e}$ by *λ* ^*k*+1^ . In (11), if let *μ* = 0, we get the classical EM algorithm. This algorithm finds all the pixel values *λ* ^*b*^. In practice, we know that *λ* should be a piecewise smooth (piecewise constant) function in PET images. However, this information is not fully utilized in the above algorithm. Below we demonstrate that such information can be incorporated and handled in an efficient way by using the level set framework, which can help to produce images with sharper edges. See also [19, 29, 30, 32–35, 40, 44] for other applications where level set based ideas are used to identify piecewise constant functions.

## 4. AN INTRODUCTION TO THE LEVEL SET METHOD

The level set method was proposed by Osher and Sethian [25] for tracing interfaces between different phases of fluid flows. Later, it has been used in many applications involving movement of interfaces for different kinds of physical problems [26–28]. In the following, we will present a “unified” framework, first presented in [29, 39, 40, 45], of using multiple level sets to represent piecewise constant functions.

Let Γ be a closed curve in Ω ⊂ ℝ^2^. Associated with Γ, we define a *ϕ* as the signed distance function:

(12) $$\phi (x)=\{\begin{array}{cc} \text{distance}(x,\Gamma ), & x\in \text{interior of}\;\Gamma ,\\ -\text{distance}(x,\Gamma ), & x\in \text{exterior of}\;\Gamma , \end{array}$$

where **x** denotes (*x, y*). It is clear that Γ is the zero level set of the function *ϕ*. In the case that Γ is not closed, but divides the domain into two parts, the level set function can be defined to be positive on one side of the curve and negative on the other side of the curve.

Once the level set function is defined, we can use it to represent general piecewise constant functions. For example, assuming that *λ*(*x, y*) equals *c*_1_ inside Γ and equals *c*_2_ outside *Γ*, it is easy to see that *λ* can be represented as

(13) $$\lambda =c_{1}H(\phi )+c_{2}(1-H(\phi )),$$

where the Heaviside function *H*(*ϕ*) is defined by

(14) $$H(\phi )=\{\begin{array}{cc} 1, & \phi >0,\\ 0, & \phi \leq 0. \end{array}$$

In order to identify the piecewise constant function *λ*, we need to identify the level set function *ϕ* and the constants *c*_i_, *i* = 1, 2.

If the function *λ*(*x, y*) has many pieces, we need to use multiple level set functions. We follow the ideas of [29, 39, 40, 46]. Assume that we have two closed curves Γ_1_ and Γ_2_, and we associate the two level set functions *ϕ*_*j*_, *j* = 1, 2 with these curves. The domain Ω can now be divided into four parts:

(15) $$\begin{array}{c} \Omega_{1}=\{x\in \Omega ,\phi_{1}>0,\;\phi_{2}>0\},\\ \Omega_{2}=\{x\in \Omega ,\phi_{1}>0,\;\phi_{2}<0\},\\ \Omega_{3}=\{x\in \Omega ,\phi_{1}<0,\;\phi_{2}>0\},\\ \Omega_{4}=\{x\in \Omega ,\phi_{1}<0,\;\phi_{2}<0\}. \end{array}$$

Using the Heaviside function again, the following formula can be used to express a piecewise constant *λ* with up to four constant values:

(16) $$\begin{array}{ll} \lambda & =c_{1}H(\phi_{1})H(\phi_{2})+c_{2}H(\phi_{1})(1-H(\phi_{2}))\\ & +c_{3}(1-H(\phi_{1}))H(\phi_{2})+c_{4}(1-H(\phi_{1}))(1-H(\phi_{2})). \end{array}$$

By generalizing, we see that *n* level set functions give the possibility of 2^*n*^ regions. For *i* = 1, 2,…, 2^*n*^, let

(17) $$bin(i-1)=(b_{1}^{i},b_{2}^{i},\ldots ,b_{n}^{i})$$

be the binary representation of *i* − 1, where $b_{j}^{i}=0$ or 1. A piecewise constant function *λ* with constant coefficients *c*_i_, *i* = 1, 2,…, 2^*n*^, can be represented as (cf. [29, 40])

(18) $$\lambda =\sum_{i=1}^{2^{n}}c_{i}\prod_{j=1}^{n}R_{i}(\phi_{j}),$$

where

(19) $$R_{i}(\phi_{j})=\{\begin{array}{cc} H(\phi_{j}), & \text{if}b_{j}^{i}=0,\\ 1-H(\phi_{j}), & \text{if}b_{j}^{i}=1. \end{array}$$

Even if the true *λ* needs less than 2^*n*^ distinct regions, we can still use *n* level set functions since some subdomains are allowed to be empty. Using such a representation, we only need to determine the maximum number of level set functions that should be utilized.

## 5. A LEVEL SET EM ALGORITHM (LSEM)

In this section, we will use the level set idea to represent *λ* as a function that only takes a limited number of constant values. Assume that the domain Ω can be divided into a union of subregions such that all *λ*_b_ have the same constant intensity value in each of the subregions. For such a case, we can use level set functions to express *λ* = *λ*(*ϕ*) as in (18). Concerning the optimization problem, we utilize the fact that calculations from (10) can be carried forward by the chain rule for *λ*(*ϕ*). As the *λ* function is already discretized, we will also use discretized level set functions *ϕ*_*j*_, *j* = 1, 2,…, *n*. From the chain rule, see [29], we get

(20) $$\begin{array}{c} \frac{\partial L}{\partial \phi_{j}}=\frac{\partial L}{\partial \lambda }\frac{\partial \lambda }{\partial \phi_{j}},\\ \frac{\partial L}{\partial c_{j}}=\int_{\Omega }\frac{\partial L}{\partial \lambda }\frac{\partial \lambda }{\partial c_{j}}. \end{array}$$

The calculation of *∂* *L*/*λ* *∂* is given in (10). We only need to have *λ* *∂*/*λ* *ϕ*_*j*_ in order to get*∂* *L*/*λ* *ϕ*_*j*_ . If *λ* takes only two constant values *c*_1_ and *c*_2_ as in (13), it is easy to see that

(21) $$\frac{\partial \lambda }{\partial \phi }=(c_{1}-c_{2})\delta (\phi ),$$

where the delta function *δ*(*ϕ*) = *H*′ (*ϕ*). In case that we need two level set functions *ϕ*_1_ and *ϕ*_2_, it follows from (16) that

(22) $$\begin{array}{c} \frac{\partial \lambda }{\partial \phi_{1}}=((c_{1}-c_{2}-c_{3}+c_{4})H(\phi_{2})+c_{2}-c_{4})\delta (\phi_{1}),\\ \frac{\partial \lambda }{\partial \phi_{2}}=((c_{1}-c_{2}-c_{3}+c_{4})H(\phi_{1})+c_{3}-c_{4})\delta (\phi_{2}). \end{array}$$

The calculation of *∂*, *λ*/*∂* *c*_*j*_ *j* = 1, 2,…, *n*, is straightforward.

For level set methods, it is standard to use the length of the level set curves as the regularization term (cf. [39, 46]). So we replace the regularization term *α* ∫_Ω_ ∣∇ *λ*∣ *d,x* in (9) by the length term $\alpha \sum_{j=1}^{n}\int_{\Omega }|\nabla H(\phi_{j})|dx$, and its derivative with respect to *ϕ*_*j*_ is the curvature −*α* ∇ ⋅(∇*ϕ*_*j*_/∣∇*ϕ*_*j*_∣)δ, where (*ϕ*_*j*_) *α* is a parameter to be used to control the influence of the regularization. Once the gradient *∂* *L*/*∂* *ϕ*_*j*_ is available, we can use the following gradient method (Algorithm 1 below) to find a minimizer for the optimization problem.

**Algorithm (level set EM algorithm).**

- Choose initial values for $\phi_{j}^{0}$ and the time step $\Delta t_{j}^{0}$.
- For all the level set functions *ϕ*_*j*_, update the functions
  (23) $$\phi_{j}^{n+1}=\phi_{j}^{n}-\Delta t_{j}^{n}\frac{\partial L}{\partial \phi_{j}}(\phi_{j}^{n}).$$
- Update the constants *c*_*j*_ :
  (24) $$c_{j}^{n+1}=c_{j}^{n}-\theta_{j}\frac{\partial L}{\partial c_{j}}.$$
- Reinitialize the level set functions *ϕ*_*j*_ if a “sufficient” amount of pixel values of *ϕ*_*j*_ have changed sign. Otherwise, go to the next iteration.

Some remarks about the implementation of the algorithm are given as follows:

- We are restricting the class of solutions to piecewise constant functions represented by (16).
- All the step length parameters can be determined by trial and error or by using a line search method to get the optimal ones.
- The parameter *α* weights the influence of the regularization. An oscillatory curve may occur if *α* is too small, and *α* too large will deny a proper evolution of the curve. By trial and error *α* ∈ (10^−3^, 10^−4^) was found to be a proper choice for the class of PET images used in our experiments.
- For numerical purpose, we approximate the delta function and the Heaviside function by
  (25) $$\delta_{\varepsilon_{1}}(\phi_{j})=\frac{\varepsilon_{1}}{\pi (\phi_{j}^{2}+\varepsilon_{1}^{2})},\;\;H_{\varepsilon_{2}}(\phi_{j})=\frac{1}{\pi }\tan^{-1}\frac{\phi }{\varepsilon_{2}}+\frac{1}{2}.$$
  This is also standard for level set methods [39]. In our numerical examples, we found that *ε*_1_ = 0.5 *h* and *ε*_2_ = 0.5 *h* worked well, where *h* refers to the grid size.
- There are efficient numerical methods to reinitialize the level set functions, see [26, 28, 47] for details. The numerical method we have used for the reinitialization is as in [40, 47], and we reinitialize the level set functions for each 30 iterations.
- We do not update the constants for every iteration, updating once for each 5–10 iterations is sufficient.
- Our approach allows the use of prior knowledge about the constants (active levels) to improve the quality of the reconstruction. We found that a reasonable estimate for these constants could help to improve the convergence of the algorithm. In all numerical implementations, an interval [*a*_*j*_, *b*_*j*_] is chosen for each of the constants *c*_*j*_, *j* = 1, 2, 3. We assume that the minimzer for *c*_*j*_ is inside [*a*_*j*_, *b*_*j*_]. During the iterations, we project *c*_*j*_ into the interval by setting *c* = min(max(*a*_*j*_, *c*_*j*_), *b*_*j*_) .

## 6. NUMERICAL RESULTS

In this section, we report some numerical results. The EM algorithm and TV-EM algorithm are implemented and will be compared with the results achieved by our LSEM algorithm. In all the examples, the observation vectors (sinogram data) were constructed by forward projection, then scaled up to total 2 × 10^6^ counts by multiplying a constant, corrupted with Poisson noise, and finally scaled back. To quantify the quality of the reconstructed images, we calculated the root mean square error (RMSE) for the reconstruction. RMSE is defined as

(26) $$\text{RMSE}=\sqrt{\frac{∥\lambda -\hat{\lambda }{∥}_{\ell^{2}}^{2}}{n}},$$

where *λ* and $\hat{\lambda }$ are two vectors that represent the computed image and the true image, respectively, and *n* is the number of pixels of the image.

### 6.1. Without prior information

**Figure 2:**
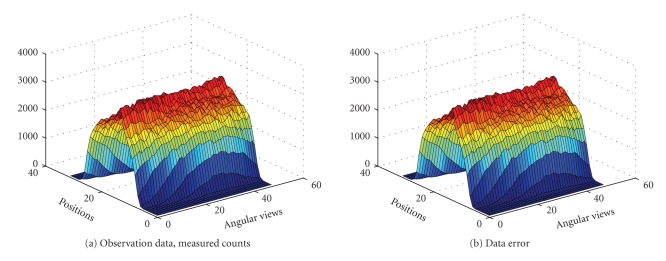
Sinogram data, obtained by forward projection: *n* = *Pλ* plus Poisson noise.

**Figure 3:**
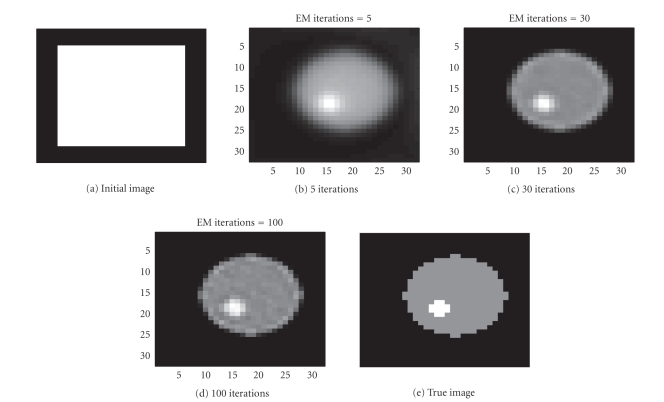
Evolution of a two circles using the EM algorithm.

In our first example we try to reconstruct a 32 × 32 image of two circles, one inside the other, as seen in Figure 3(e). Total 1536 (32 positions and 48 angular views) observations were given to us. The sinogram data as well as the data noise (after scaling up) are shown in Figure 2. The true intensity values are {0, 1, 2}. We first test the EM algorithm. After a few iterations, it is possible to see some inner structures in the PET image depicted in Figure 3.

**Figure 4:**
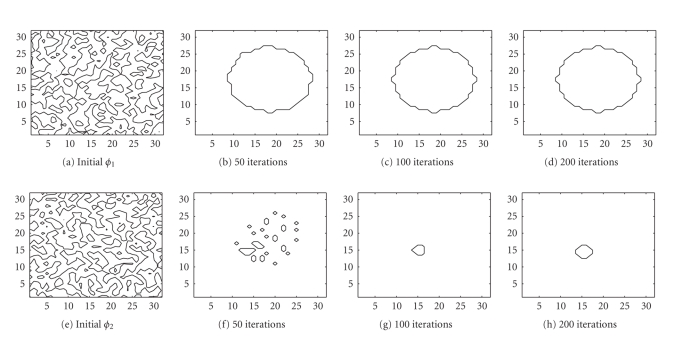
Interfaces given by the zero level set of the function *ϕ*_1_ and *ϕ*_2_.

The major drawback with the EM algorithm is its lack of termination criterion and the introduction of noise as the number of iterations increases. In Figure 3(b), the intensity values in the outer circle are almost constant (as it should be in this test), but it is difficult to decide the exact boundary for the inner circle. After 30 iterations, the edges are emphasized but so is the noise, as seen in Figure 3(c). After 100 iterations, the noise becomes dominant and degrades the quality of the recovered intensity function. The same sinogram data were thereafter used for the TV-EM and the LSEM algorithms. The results are shown in Figure 4. For the two level set functions of the LSEM algorithm, we started with random initial guesses (cf. Figures 4(a) and 4(e)).

**Figure 5:**
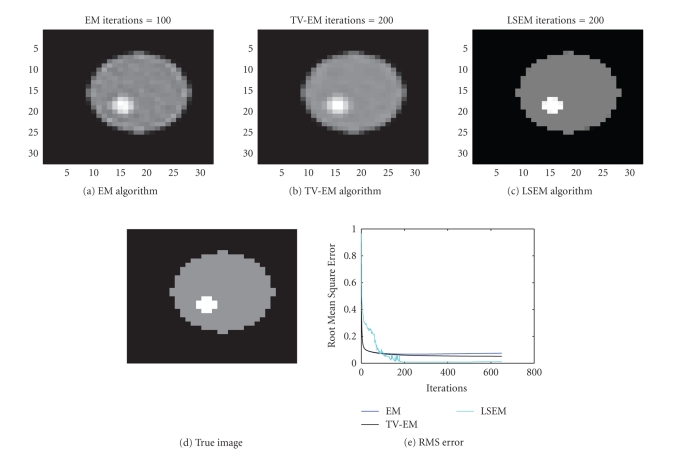
PET image of two circles constructed with different algorithms.

In less than 200 iterations, both level set functions have converged to a constant shape and these level set functions together with (16) were used to get Figure 5(c). With two level set functions, we see from (15) that it is possible to identify up to 4 distinct regions. The true PET image depicted in Figure 5(d) consists of only 3 distinct regions: background, outer circle, and inner circle. To handle this, we put *c*_1_ = *c*_3_ such that 2 regions yield the same contribution to the constructed PET image. The intervals for the intensity values are { [0, 0.5], [0.5,1.5], [1.5, 2.5] } . After 200 iterations, the intensity values are recovered as { [0, 1.0005, 2.0192 }, which match the true values very well.

Even though this is a simple test that involves a nonmedical image, it illustrates the potential in the LSEM algorithm. Sharp edges are recovered properly for the PET image and different regions do not suffer from inhomogeneities caused by noise. Notice the improvement of LSEM over EM and TV-EM in the recovery of the shape of the inner circle in Figure 5. The RMS error, as shown in Figure 5(e), also suggests that the LSEM algorithm produces the best reconstruction.

**Figure 6:**
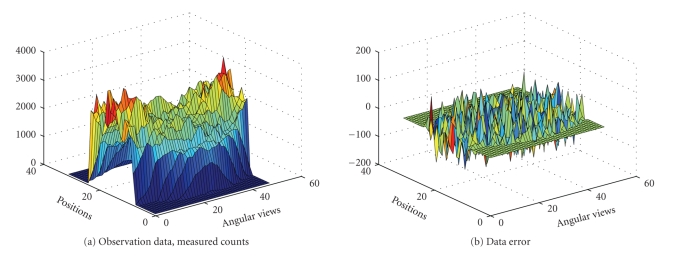
Sinogram data, obtained by forward projection: *n* = *Pλ* plus Poisson noise.

**Figure 7:**
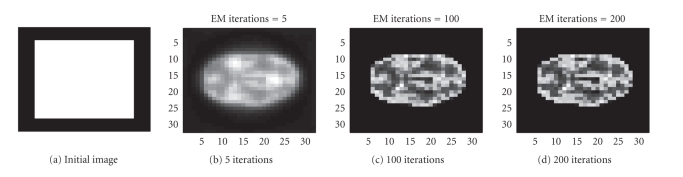
Evolution of a brain image with the EM algorithm.

In the next example, the interior structure of the PET image is more complicated. We try to reconstruct a 32 × 32; image of the brain from 1536 observations (32 positions and 48 angular views, synthetic data). The sinogram data as well as the data noise (after scalling up) are shown in Figure 6. The true intensity values are { 0,1,4}. The results obtained with the EM algorithm are displayed in Figure 7.

**Figure 8:**
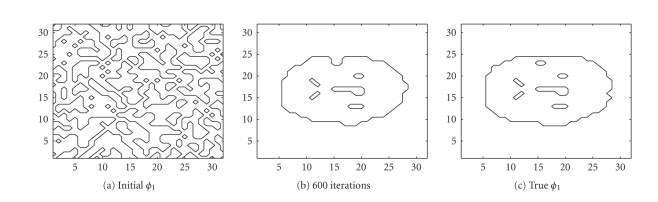
Interfaces given by the zero level set of the function *ϕ*_1_.

**Figure 9:**
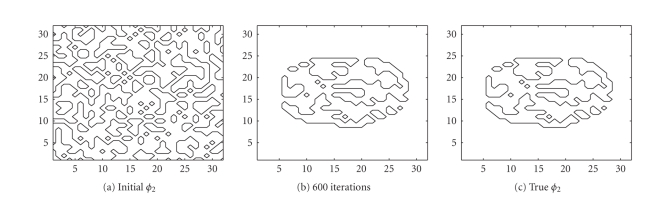
Interfaces given by the zero level set of the function *ϕ*_2_.

We also used the same sinogram data to test the TV-EM and the LSEM algorithms. For the LSEM algorithm, the evolutions of the two functions *ϕ*_1_ and *ϕ*_2_ are given in Figures 8 and 9, respectively.

**Figure 10:**
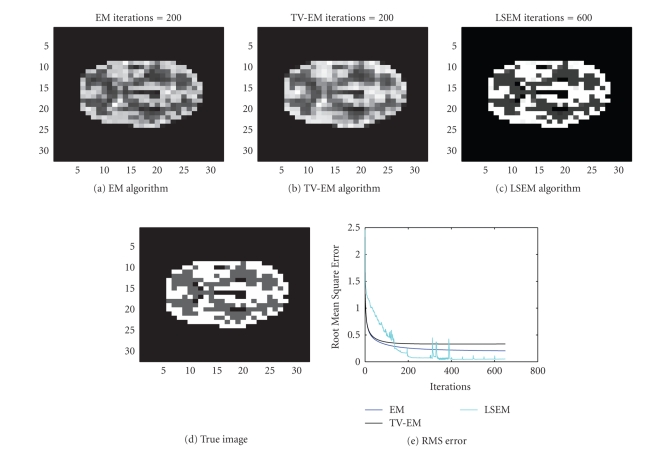
Construction of a PET image with different algorithms.

In less than 600 iterations, the two level set functions have converged. Combining *ϕ*_1_ from Figure 8(c) and *ϕ*_2_ from Figure 9(c) together with (16), we get the PET image depicted in Figure 10(c). In this test, we used *c*_4_ (background), *c*_2_ (gray matter), and *c*_1_ = *c*_3_ (white matter). The intervals for the intensity values are: { [0, 0.5], [0.5, 1.5], [3.5, 4.5] }. After 600 iterations, the intensity values are recovered pretty well as { 0, 0.9802, 4.0620 }.

In Figures 10(a) and 10(b), the boundaries between the tissue classes are not sharp. In contrast, we see that LSEM algorithm is able to recover almost all the fine details in the PET image in this example.

**Figure 11:**
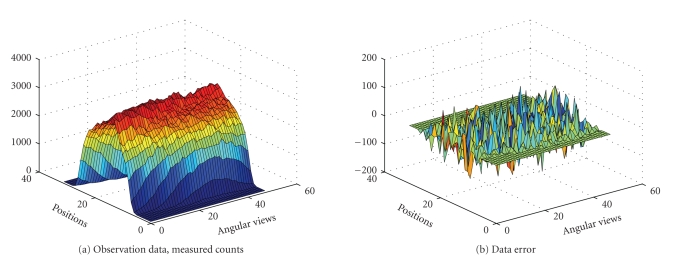
Sinogram data, obtained by forward projection: *n* = *Pλ* plus Poisson noise.

**Figure 12:**
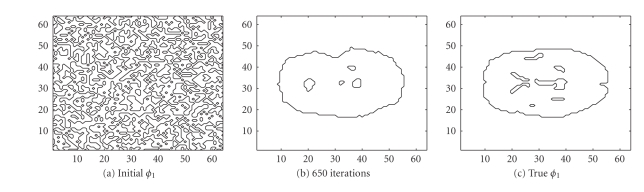
Interfaces given by the zero level set of *ϕ*_2_.

**Figure 13:**
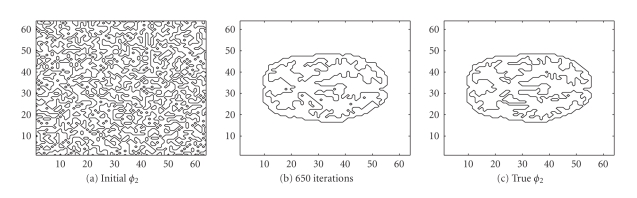
Interfaces given by the zero level set of *ϕ*_2_ .

**Figure 14:**
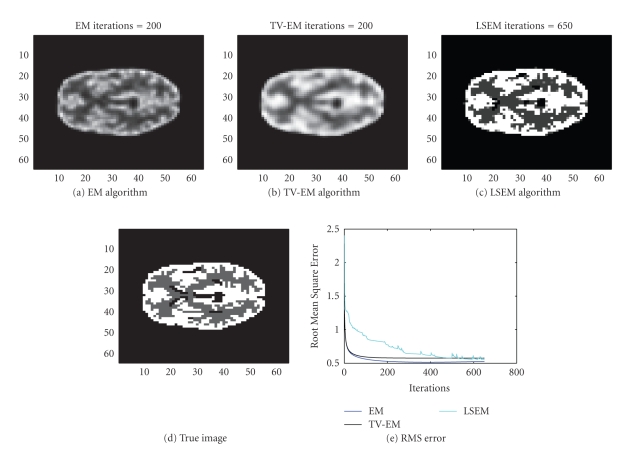
64 × 64 segmented MRI slice of the brain.

Next, we challenge our algorithm with a 64 × 64 image obtained from a segmented MRI slice of the brain. This image was used to generate totally 6144 (64 positions and 96 angular views) observations. The sinogram data as well as the data noise (after scaling up) are shown in Figure 11. Notice that we are using the MRI image to generate the PET data, and we are not trying to solve the MRI tomography problem. The true intensity values are {0, 1, 4}, and the intervals { [0, 0.5], [0.5, 1.5], [3.5, 4.5] } were used for our LSEM algorithm. Compared with Figures 5(c) and 10(c), the inner structure to be recovered here is more complicated, as seen in Figure 14(c). The evolutions of the *ϕ*_1_ and *ϕ*_2_ functions are shown in Figures 12 and 13.

We see that the level set function *ϕ*_2_ is recovered rather accurately, while the interior structure for *ϕ*_1_ is not that nicely recovered. This will influence the appearance of the PET image, as seen in Figure 14(b). After 650 iterations, the intensity values are recovered as {0, 0.95549, 3.8629}. If we look at Figure 14(e), the RMS error does not reveal any advantages for the LSEM algorithm. Even so, due to the clearly identified dark region and the sharp edges in Figure 14(c), the LSEM algorithm still produces a better result than what was achieved with the EM algorithm in Figure 14(a) or TV-EM in Figure 14(b).

### 6.2. Incorporating prior information

To obtain improved reconstructions, one approach is to use priors that reflect the nature of the underlying radionuclide distribution. Recently, there has been a considerable interest in incorporating side information derived from highly correlated anatomical information (such as MRI and CT) in the form of Bayesian priors [22, 48]. The main attraction of this approach is that one can expect to obtain improved reconstructions to the extent that functions follow anatomy. Usually, the anatomical information is incorporated by some penalty technique, and a parameter *β* is used to control the influence of the priors, which should smooth the image under reconstruction. There are also some papers dedicated to keeping sharp boundaries in the smoothing process. The key point there is to try to derive and represent the boundary information in the form of local smoother from the anatomical MRI or CT image. However, by level set formulation, the anatomical information can be incorporated into the EM algorithm in a natural and efficient way, and the sharp boundaries are preserved naturally and easily. We just need to know the location of the boundaries, the intensity values in the CT or MRI image are not necessary.

**Figure 15:**
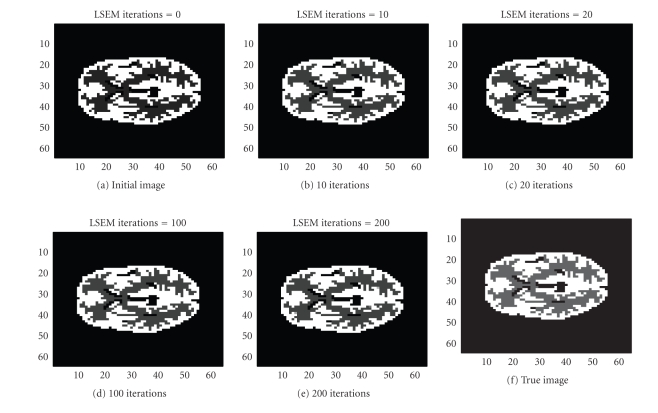
64 × 64 segmented MRI slice of the brain.

Assume that MRI or CT observations are used to generate information of the PET phantom, partly or in the entire domain Ω —see [49, 50]. Below we will demonstrate that such information will improve the image reconstruction capacity noticeably. First, we assume both *ϕ*_1_ and *ϕ*_2_ to be known, which means that all the boundaries are known a priori, and we just need to recover the piecewise constant intensity values of the image. The result is shown in Figure 15. Compared with the results in Figure 14, we see that a prior information of the geometrical objects improves the reconstruction dramatically. We need only about 20 iterations to reconstruct a perfect image. In this case, after 200 iterations, the intensity values were recovered pretty well as {0, 1.01, 3.98}.

**Figure 16:**
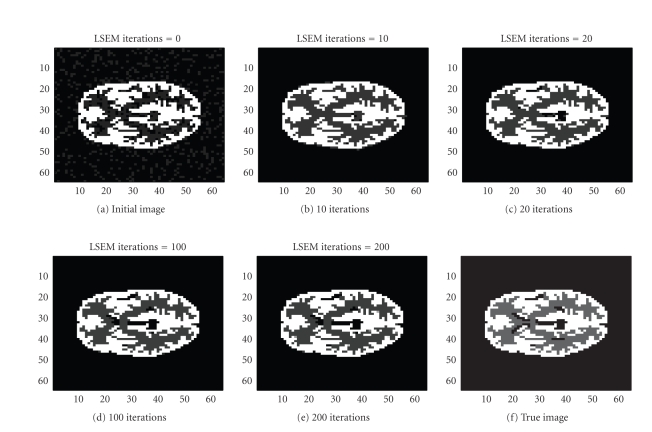
64 × 64 segmented MRI slice of the brain.

Next, we assume *ϕ*_2_ to be known, and let *ϕ*_1_ evolves freely. This corresponds to wrong or incomplete anatomical information. The results are shown in Figure 16. From this example, we see that LSEM can tolerate wrong or incomplete anatomical priors. In this case, LSEM will try to discard the wrong information. After 200 iterations, the intensity values were recovered as {0, 0.97, 3.99}.

In our final example, we try to show that our method works well for nonpiecewise constant images. Let *λ*(*x, y*) denotes the 64 × 64 true piecewise constant image as in the above example. We also assume that *ϕ*_2_ is known, while *ϕ*_1_ is unknown.

Firstly, we add a smooth function to the original piecewise constant image, so that the true image is somehow piecewise smooth,

(27) $$\bar{\lambda }(x,y)=\lambda (x,y)+\sigma ⋆(\sin(16\pi x)\sin(16\pi y)).$$

**Figure 17:**
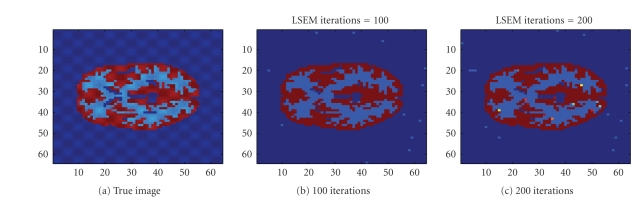
64 × 64 segmented MRI slice of the brain, nonpiecewise constant by adding sin() functions.

The original true image as well as the reconstructed image are shown in Figure 17. We use *σ* = 0.1 in this test. The intensity values were recovered as {0, 0.92, 3.99}.

Then, we add random noise to the true piecewise constant image by

(28) $$\bar{\lambda }(x,y)=\lambda (x,y)+\sigma ⋆(Rand(x,y)-0.5),$$

where Rand (*x, y*) produces uniformly distributed real numbers between [0, 1]. In this test, we use *σ* = 0.2.

The original true image as well as the reconstructed image are shown in 18. The intensity values were recovered as {0, 0.99, 3.99}.

## 7. CONCLUSIONS

We have applied a level set method to the positron emission tomography reconstruction problem, based on the assumption that the active values can be identified with different levels.

The basic idea is to modify the maximum likelihood expectation maximization algorithm by using a level set formulation. With this approach, we can incorporate anatomical prior information naturally and easily into the algorithm.

## Figures and Tables

**Figure 18:**
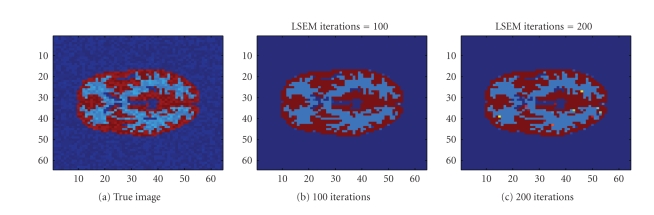
64 × 64 segmented MRI slice of the brain, nonpiecewise constant by adding Rand() functions.

**Table 1:** Notations used throughout this paper.

*b*	Pixel index (1, 2, … ,*B*

*λ_b_*	Unknown source intensity at a pixel *b*, *λ_b_* ≥ 0 for all *b*

*t*	Detector pair index (1, 2, …, *T*

*n_t_*	Total number of coincidences counted by detector pair *t*, *n_t_* ≥ 0 for all *t*

*P_tb_*	Probability for an emission from *b* to be detected in *t*
